# Rapid Determination of Major Compounds in the Ethanol Extract of Geopropolis from Malaysian Stingless Bees, *Heterotrigona itama*, by UHPLC-Q-TOF/MS and NMR

**DOI:** 10.3390/molecules22111935

**Published:** 2017-11-10

**Authors:** Lingling Zhao, Mengjiao Yu, Minghui Sun, Xiaofeng Xue, Tongtong Wang, Wei Cao, Liping Sun

**Affiliations:** 1 Institute of Apicultural Research, Chinese Academy of Agricultural Sciences, Beijing 100093, China; yixian111624@126.com (L.Z.); mengjiaoyu2018@126.com (M.Y.); smh460112@126.com (M.S.); 2 Institute of Analytical Science, Shaanxi Provincial Key Lab of Electroanalytical Chemistry, Northwest University, Xi’an 710069, China; caowei@nwu.edu.cn; 3 Institute of Quality Standard and Testing Technology for Agro–Products, Beijing 100081, China; wangttong123@126.com

**Keywords:** geopropolis, UHPLC-Q-TOF/MS, stingless bees, *Heterotrigona itama*

## Abstract

A reliable, rapid analytical method was established for the characterization of constituents of the ethanol extract of geopropolis (EEGP) produced by Malaysian stingless bees—*Heterotrigona itama*—by combining ultra-high-performance liquid chromatography with quadruple time-of-flight mass spectrometry (UHPLC-Q-TOF/MS). Based on known standards, the online METLIN database, and published literature, 28 compounds were confirmed. Phenolic acids, flavones, triterpenes and phytosterol were identified or tentatively identified using characteristic diagnostic fragment ions. The results indicated that terpenoids were the main components of EEGP, accompanied by low levels of phenolic acids, flavonoids, and phytosterol. Two major components were further purified by preparative high-performance liquid chromatography (PHPLC) and identified by nuclear magnetic resonance (NMR) as 24(*E*)-cycloart-24-ene-26-ol-3-one and 20-hydroxy-24-dammaren-3-one. These two triterpenes, confirmed in this geopropolis for the first time, are potential chemical markers for the identification of geopropolis from Malaysian stingless bees, *H. itama*.

## 1. Introduction

Geopropolis is a colloidal solid produced by stingless bees that is composed of resin collected from various plants together with wax secretions, mud and sand [1,2]. Similar to *Apis mellifera* propolis, geopropolis is used for building honeycomb and for the maintenance of bee health. However, geopropolis differs from *A. mellifera* propolis in that it includes wax and soil in its composition, giving it special characteristic features. The complex chemical composition of geopropolis determines its diverse bioactivities. Geopropolis preparations have long been used in wound repair, for the treatment of digestive, respiratory, skin and vision disorders, and as antimicrobial agents and preservatives [2,3,4]. For example, geopropolis produced by *Melipona fasciculata* Smith from Brazil exhibits antimicrobial activity against *Streptococcus mutans*, *Lactobacillus acidophilus* and *Candida albicans*, giving it potential as a drug for the prevention or control of oral cavity infections [5]. Geopropolis produced by *M. compressipes fasciculata* Smith exerts antibacterial activity against *S. mutans* isolated from the human oral cavity [6]. Ethanolic extract of geopropolis (EEGP) from *Melipona scutellaris* exhibits antimicrobial activity against *Staphylococcus aureus*, *S. mutans*, and methicillin-resistant Staphylococcus aureus (MRSA) strains [7]. Geopropolis was found to exert fungistatic activity towards *Pythium insidiosum* rather than a fungicidal effect, when compared with propolis [4]. Geopropolis has been found to have antitumoral and immunomodulatory activity, and was cytotoxic towards canine osteosarcoma cells [8]. Geopropolis has also been found to be cytostatic towards human laryngeal epidermoid carcinoma cells and is known to stimulate tumor necrosis factor alpha (TNF-α) and interleukin-10 (IL-10) production by human monocytes. It was cytotoxic to monocytes only at its highest concentration, while at non-cytotoxic concentrations it increased TNF-α and IL-10 production by these cells. This pharmacological property of geopropolis may be due to triterpenes, which are some of its major chemical constituents [9]. EEGP from *M. scutellaris* and its aqueous fraction decreased the migration of neutrophils in the inflammatory process, and this was dependent on the nitric oxide pathway [10].

The diverse biological properties and wide application of geopropolis in modern medicine have meant increasing attention has been paid to the identification of new sources of geopropolis and to the study of their chemical composition. Recently, a form of geopropolis produced by stingless bees (*Heterotrigona itama*) and collected in the state of Sarawak, Malaysia, has been shown to exhibit antibacterial activity as well as antioxidant, nitric oxide scavenging, and antidiabetic activities [11,12].

The chemical constituents of this geopropolis have been tentatively studied based on thin layer chromatography and color reactions [11,12]. The results showed that its methanol extract was composed of terpenoids, flavonoids, phenols, steroids, saponin, and coumarins. However, detailed information on all components—including structural characterization—is not available. Identification and characterization of geopropolis components is, therefore, essential for the further study of its pharmacological activity and toxicology.

It is not possible to rapidly identify all components of a complex mixture using traditional identification methods such as isolation, purification, mass analysis, NMR and IR analysis. In order to quickly identify compounds in complex product mixtures, some new methods have been developed. LC-MS/MS or LC-Q-TOF-MS combined with database and MS fragmentation analysis is an emerging technology that is widely used to analyze complex samples in order to provide possible molecular formulas and reliably identify unknown compounds. It has been used for the analysis of Chinese traditional medicines [13], propolis [14] and plant extracts [15]. It is generally difficult to identify highly polar triterpenoids, flavonols and siraitic acid glycosides using conventional phytochemical methods, so it is necessary to identify these chemicals by LC-Q-TOF /MS [16].

In the present study, the components of the ethanol extract of geopropolis produced by *H. itama* were analyzed using ultra-high-performance liquid chromatography with quadruple time-of-flight mass spectrometry (UHPLC-Q-TOF/MS), target MS/MS data acquisition strategy. Consequently, aided by molecular feature extraction using an Agilent MassHunter Workstation, Agilent Molecular Structure Correlator (MSC) software, the free online database METLIN, and fragmentation pathway rules determined from reference compounds, 28 compounds were identified or tentatively identified. This comprehensive research on geopropolis could provide a meaningful basis for further quality control, pharmacological studies, and toxicological research.

## 2. Results and Discussion

**Figure 1:**
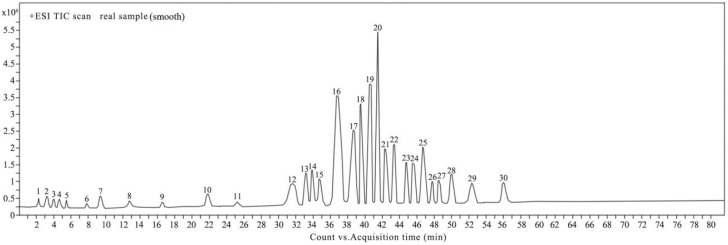
The total ion chromatograms from ultra-high-performance liquid chromatography with quadruple time-of-flight mass spectrometry (UHPLC-Q-TOF/MS) in positive mode.

For the identification of unknown compounds in natural products, first a known standards database is usually built and then used to match unknown compounds. In this study, we collected 26 active compound standards including phenolic acids, flavonoids and their derivatives, which had been reported as present in propolis or geopropolis. We then established a UHPLC-Q-TOF/MS method for analyzing the 26 compounds for a comparison with the compounds in EEGP. The separation of the constituents was performed by an Agilent ZORBAX SB-Aq C_18_ column, which is suitable for the high polar compounds and high percentage of the aqueous phase, and with which excellent separation and symmetry peak shapes can be obtained. This was successfully applied to the characterization of the constituents of EEGP (Figure 1). Consequently, 30 compounds—including phenolic acids, flavonoids, naphthoquinones, triterpenes and phytosterol—were either identified based on the known standards and NMR, or tentatively identified using characteristic diagnostic fragment ions and literature data.

### 2.1. Identification of Compounds from EEGP by UHPLC-Q-TOF/MS

#### 2.1.1. Identification of Compounds in EEGP Based on Known Authentic Standards

By comparing the retention times with the accurate mass spectra of the standards, several phenolic acids, such as gallic acid (peak 1), caffeic acid (peak 2), syringic acid (peak 9), and benzoic acid (peak 12), were identified in EEGP. It has been reported that gallic acid [17], caffeic acid [18], and cinnamic acid [19] were found in *Tetragonisca angustula* geopropolis from Brazil. Benzoic acid and syringic acid were found for the first time in geopropolis from *H. itama* and these phenolic acids were found in EEGP by comparison with known standards.

Pinobanksin (peak 16) and kaempferol (peak 18) were also detected in EEGP. Since stingless bee species have differing preferences for various propolis plants, few flavonoids are found in geopropolis and these are at low levels. Some flavonoids—including catechin, kaempferol and morin—were found in geopropolis from Brazil [20]. 7-*O*-methyl-naringenin (*Melipona subnitida*) [21], (2S)-pinostrobin (*Tetragonula carbonaria*) and other dihydroflavanones [22] were identified in Brazilian and Australian geopropolis. The presence of flavonoid glycosides such as rutin has also been reported [20]. This study is the first report of pinobanksin in geopropolis, and this flavone was found in EEGP by comparison with known standards.

#### 2.1.2. Identification of Compounds in EEGP using METLIN and MSC Software

Based on the molecular feature extraction using the Agilent MassHunter Workstation, all compounds (*m*/*z*) were first extracted from the total ion current (TIC) chromatogram and saved in “cef” format. All data were then loaded into the MSC software and the online METLIN database was searched for potential matches.

As shown in Table 1, peak 2 was also identified as caffeic acid using the MSC software. The MSC software showed a conducted experiment to investigate the fragmentation behavior of gallic acid. In negative mode, the [M − H]^−^ ion was at *m*/*z* 169.0144 (C_7_H_5_O_5_). In the negative MS/MS spectrum, a characteristic fragment ion at *m*/*z* 125.0234 (C_6_H_5_O_3_) could be deduced to represent loss of a −COO unit. This loss of 44 Da (−COO) could be considered characteristic fragmentation behavior of a phenolic acid. An additional fragment ion at *m*/*z* 107.0128 (C_6_H_3_O_2_) could be attributed to loss of neutral water (loss of 18) via the adjacent phenolic hydroxyl unit.

Other compounds—including gallic acid (peak 1) and benzoic acid (peak 12)—were also tentatively identified using the MSC software. This result was consistent with that obtained using benzoic acid, gallic acid, and caffeic acid authentic standards as reference materials, confirming that the MSC software is an effective tool for the tentative identification of unknown compounds.

The molecular formula of peak 4 could be deduced as C_14_H_20_O_9_ from the [M − H]^−^ ion at *m*/*z* 331.1031. In the negative MS/MS spectrum, a dominant fragment ion at *m*/*z* 169.0133 (C_7_H_5_O_5_) represented a loss of 162 Da, which could be tentatively attributed to a hexose unit. Another prominent ion at *m*/*z* 125.0232 (C_6_H_5_O_3_), obtained by Q-TOF analysis, was assigned as a loss of −COO (44 Da) from *m*/*z* 169.0133. In addition, the fragment ion at *m*/*z* 125.0232 could lose H_2_O (18 Da) directly to produce an ion at *m*/*z* 107.0127 (C_6_H_2_O_2_). According to the fragmentation behavior, peak 4 was identified as gallic acid-hexose.

Peak 5 gave a [M − H]^−^ ion at *m*/*z* 167.0362 (C_8_H_7_O_4_) and a [M + H]^+^ ion at *m*/*z* 169.0491. It produced a fragment ion at *m*/*z* 153.0206 (C_7_H_5_O_4_) by loss of 14 Da, attributed to loss of a −CH_2_ group, and an ion at *m*/*z* 123.0452 (C_7_H_7_O_2_) by loss of 44 Da (loss of −COOH group). Peak 5 could therefore be tentatively identified as vanillic acid.

Peak 6, with the same fragment ions as peak 5, could be tentatively identified as an isomer of vanillic acid.

Peak 7 was tentatively identified as caffeine, having a precursor ion at *m*/*z* 195.0877 (C_8_H_11_N_4_O_2_), and fragment ions at *m*/*z* 150.0888 [M + H − 45 Da], 138.0668 [M + H − 45 − 12 Da], and 110.0713 [M + H − 45 − 12 − 28 Da].

Peak 10 gave precursor ions [M − H]^−^ at *m*/*z* 153.0192 (C_7_H _5_O_4_) and [M + H]^+^ at *m*/*z* 155.0344. In negative MS/MS mode fragment ions were observed at *m*/*z* 123.0416 (C_6_H_3_O_3_) by the loss of 30 Da, attributed to the loss of a H_2_C=O group, and at *m*/*z* 109.0285 (C_6_H_5_O_2_), attributed to the elimination of a −COO group. Ions at *m*/*z* 153.0192, 123.0416 and 109.0285 are diagnostic ions for protocatechuic acid.

Peak 11, tentatively identified as pyrogallol, produced precursor ions [M − H]^−^ at *m*/*z* 125.0244 (C_6_H_6_O_3_) and [M + H]^+^ at *m*/*z* 127.0361. In negative MS/MS mode and at different collision energies, only one fragment ion was produced at *m*/*z* 107.0130 [M−H − 18 Da] (C_6_H_4_O_2_), which was consistent with its chemical structure.

Peak 13 was tentatively identified as vitexin-*O*-gallate, with an [M − H]^−^ ion at *m*/*z* 583.1118 (C_28_H_24_O_14_). A characteristic ion at *m*/*z* 431.0997 (C_21_H_19_O_10_) by the loss of a hexose (162 Da) enabled tentative identification of vitexin. The ions at *m*/*z* 313.0577 (C_13_H_12_O_9_) and *m*/*z* 269.0641 (C_12_H_12_O_7_) were attributed to subsequent successive losses of C_8_H_5_O and CO_2_. Another fragment ion at *m*/*z* 169.0142 could be tentatively assigned as gallic acid, from which *m*/*z* 125.0234 could be deduced to represent the loss of a −COO unit.

Peak 14, with the same fragment ions as peak 13, could be tentatively identified as an isomer of vitexin-*O*-gallate.

Peak 19 produced ions [M − H]^−^ at *m*/*z* 247.0942 (C_14_H_15_O_4_) and [M + H]^+^ at *m*/*z* 249.1123 (C_14_H_17_O_4_). In positive MS/MS mode, characteristic peak ions were observed at *m*/*z* 217.0884 (C_13_H_12_O_3_), 172.0088 (C_12_H_12_O_1_) and 144.0939 (C_11_H_12_) after the successive loss of CO_2_ and one neutral molecular CO group. The compound was tentatively identified as prenyl caffeate.

Peak 24 produced precursor ions [M + H]^+^ at *m*/*z* 451.1506 and [M − H]^−^ at *m*/*z* 449.1462 (C_22_H_26_O_10_). In the negative MS/MS spectrum, diagnostic fragment ions corresponding to the elimination of −CH_3_CH_2_O, hexose, −CH_3_, and −CO at *m*/*z* 407.1322, 245.0482, 230.0665 and 202.0516 were observed. Ultimately, peak 24 was tentatively confirmed to be torachrysone-*O*-(acetyl)-hexose.

In addition to the aforementioned major components, several minor constituents were identified including acetyleugenol (peak 15), umbelliferone (peak 17), lapachol (peak 21), torachrysone-*O*-hexose (peak 23), mangostin (peak 26), ganoderol A (peak 27), saringosterol (peak 28), stigmasterol (peak 29), and taraxerone (peak 30). Their likely structures were determined by reference to known compounds from EEGP and comparison of their mass spectra with literature data. The MS and MS/MS data are provided in Table 1.

### 2.2. Identification of Unknown Compounds using Preparative HPLC (PHPLC) and NMR

As seen in Figure 1, there were two strong peaks with retention times of 22–24 min (peak 20, peak 22), which could not be tentatively confirmed using the MSC software and the METLIN database. These two compounds were purified by PHPLC and their NMR spectra were analyzed.

The molecular formula, molecular weight, ^13^C-NMR and ^1^H-NMR spectroscopic data for peak 20 are presented below.

Peak 20 showed an [M − H]^−^ ion at *m*/*z* 439.3583 (C_30_H_48_O_2_) and [M + H]^+^ at *m*/*z* 441.3725.

^1^H-NMR (400 MHz, CDCl_3_) δ: 0.58 (d, 1H, *J* = 4.3 Hz, 19-H), 0.79 (d, 1H, *J* = 3.8 Hz, 19-H), 1.68 (s, 3H, 27-H), 1.11 (s, 3H, 29-H), 1.05 (s, 3H, 28-H), 1.00 (s, 3H, 18-H), 0.91 (s, 3H, 30-H), (s, 3H, 27-H), 2.21 (dm, 1H, *J* = 14.1, 2-H), 2.72 (td, 1H, *J* = 14.1, 6.5 Hz, 2-H), 4.01 (d, 2H, *J* = 5.8 Hz, 26-H), 5.41 (t, 1H, *J* = 5.4 Hz, 24-H).

^13^C-NMR (100 MHz, CDCl_3_) δ: 216.6 (3-C), 134.3 (25-C), 127.0 (24-C), 69.1 (26-C), 52.3 (17-C), 50.2 (6-C), 48.7 (14-C), 48.4 (5-C), 47.9 (8-C), 45.3 (13-C), 37.5 (2-C), 35.9 (15-C), 35.9 (20-C), 35.5 (22-C), 33.4 (1-C), 32.8 (12-C), 29.5 (19-C), 28.1 (7-C), 26.7 (11-C), 26.0 (10-C), 25.9 (23-C), 24.5 (16-C), 22.2 (28-C), 21.5 (6-C), 21.1 (9-C), 20.8 (29-C), 19.3 (30-C), 18.2 (21-C), 18.1 (18-C), 13.6 (27-C).

**Figure 2:**
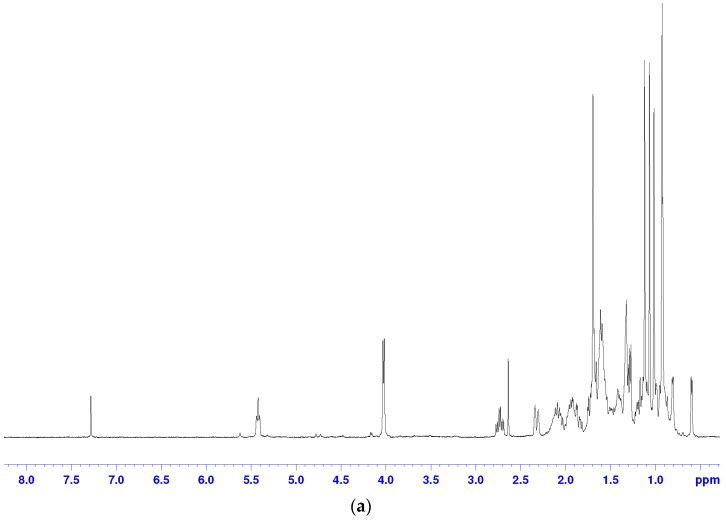

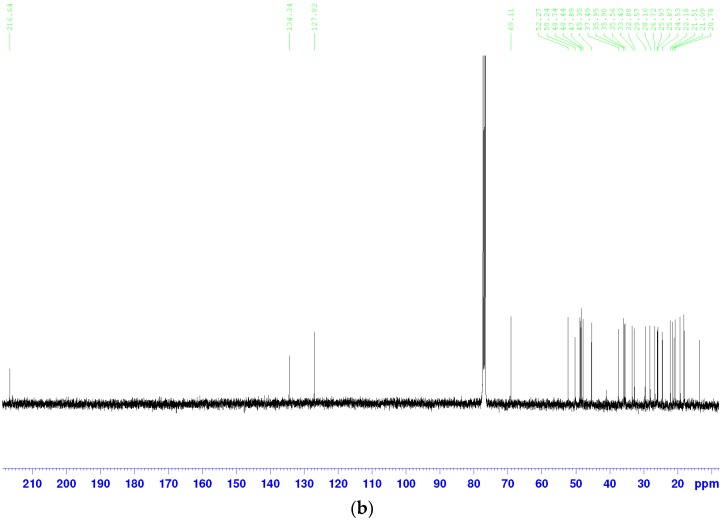
(**a**) ^1^H-NMR and (**b**) ^13^ C-NMR spectral data of 24(*E*)-cycloart-24-ene-26-ol-3-one.

**Figure 3:**
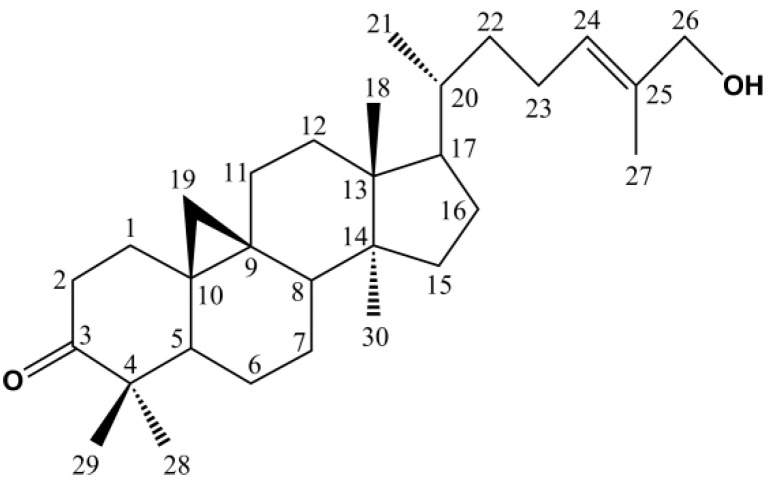
The structure of 24(*E*)-cycloart-24-ene-26-ol-3-one.

^1^H-NMR and ^13^C-NMR Spectra Are Shown in Figure 2. Based on NMR data and the literature [23], peak 20 was identified as 24(*E*)-cycloart-24-ene-26-ol-3-one, and its structure is presented in Figure 3. This compound was reported to have anti-cancer potential without the adverse effects observed with TNF-α, suggesting that further development of this cycloartane as an anti-cancer drug was worthwhile. This implies that geopropolis produced by *H. itama* may be useful as a raw material for the production of anti-cancer drugs in the future.

The molecular formula, molecular weight, ^13^C-NMR and ^1^H-NMR spectroscopic data for peak 22 are presented below.

Peak 22 showed an [M − H]^−^ ion at *m*/*z* 441.3736 (C_30_H_50_O_2_) and [M + H]^+^ at *m*/*z* 443.3882.

^1^H-NMR (400 MHz, CDCl_3_) δ: 0.89 (30-H), 0.94 (18-H), 1.00 (s, 3H, 19-H), 1.04 (s, 3H, 29-H), 1.08 (s, 3H, 28-H), 1.15 (s, 3H, 21-H), 1.63 (s, 3H, 26-H), 1.69 (s, 3H, 27-H), 2.21 (ddd, 1H, *J* = 15.6, 7.8, 3.3 Hz, 2-H), 2.52 (ddd, 1H, *J* = 15.8, 9.0, 1.3 Hz, 2-H), 5.12 (t, 1H, *J* = 6.5 Hz, 24-H).

^13^C-NMR (100 MHz, CDCl_3_) δ: 218.1 (3-C), 131.6 (25-C), 124.7 (24-C), 75.3 (20-C), 55.3 (5-C), 50.2 (14-C), 50.0 (9-C), 49.8 (17-C), 47.4 (4-C), 42.4 (13-C), 40.4 (22-C), 40.2 (8-C), 39.9 (1-C), 36.8 (10-C), 34.5 (7-C), 34.1 (2-C), 31.1 (15-C), 27.5 (16-C), 26.7 (28-C), 25.7 (26-C), 25.5 (12-C), 24.8 (21-C), 22.5 (23-C), 22.0 (11-C), 21.0 (29-C), 19.6 (6-C), 17.7 (27-C), 16.3 (30-C), 16.0 (18-C), 15.2 (19-C).

**Figure 4:**
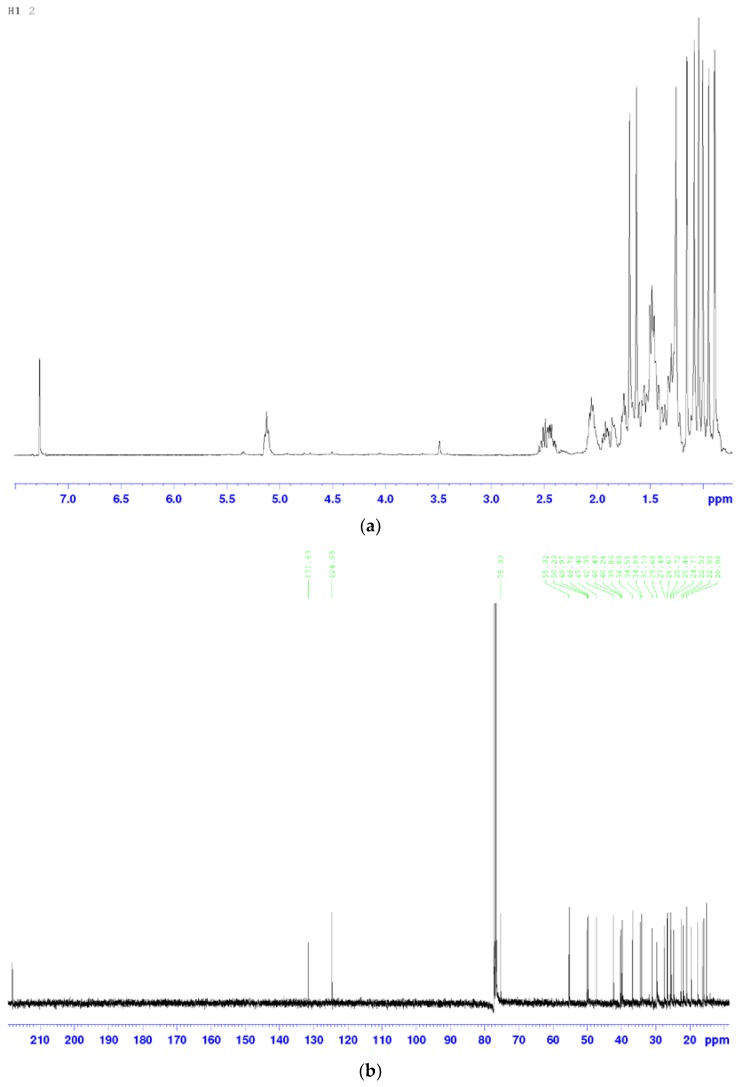
(**a**) ^1^H-NMR and (**b**) ^13^C-NMR spectral data of 20-hydroxy-24-dammaren-3-one.

**Figure 5:**
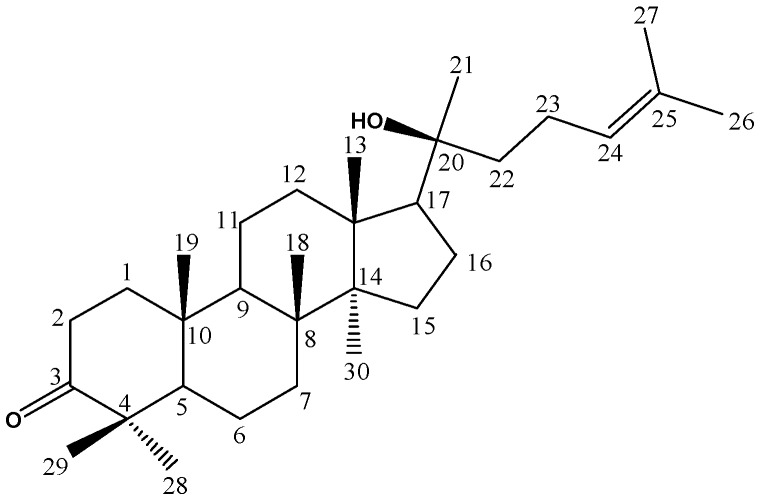
The structure of 20-hydroxy-24-dammaren-3-one.

^1^H-NMR and ^13^C-NMR Spectra Are Shown in Figure 4. Based on NMR data and the literature [24], peak 22 was identified as 20-hydroxy-24-dammaren-3-one, and its structure is presented in Figure 5. This triterpenoid compound has previously been extracted from the stem bark of *Toona sinensis* [24].

From the abundance of peaks in the TIC, it can be concluded that terpenoids are the main components of EEGP, while low levels of phenolic acids, flavonoids and phytosterol are present. Terpene compounds are the main active components of geopropolis. There have been a number of reports on terpenoids in geopropolis [18,19,25,26,27,28,29]. Monoterpenes such as limonene [27] were detected in Mexican geopropolis—δ-cadinene [26] and other sesquiterpenes were identified in Bolivian geopropolis. Massaro FC [19] identified diterpenoids—such as abietic acid—in *T. carbonaria* geopropolis. In respect of triterpenes, there are reports that cycloartenol [18], dipterocarpol [28] and santolinatriene [29] have been found in Brazilian, Thai and Mexican geopropolis.

In the present study, we identified two abundant terpenoids in EEGP—24(*E*)-cycloart-24-ene-26-ol-3-one and 20-hydroxy-24-dammaren-3-one. Published research has demonstrated that these two terpenoids have biological activity [23,24]. The confirmation of their presence in geopropolis produced by *H. itama* makes these compounds potential markers for this geopropolis.

## 3. Materials and Methods

### 3.1. Chemicals

HPLC grade methanol and formic acid were purchased from Merck Technologies Inc. (Darmstadt, Germany). Deionized water was obtained from a Millipore Milli-Q water system (Bedford, MA, USA). All other reagents were of analytical purity. Geopropolis samples produced by *H. itama* were collected from the state of Sarawak, Malaysia and were identified by Professor Yi-Lin Sophia Chen (Department of Biotechnology and Animal Science, National Ilan University, Taiwan). A voucher specimen was deposited in the local laboratory of R H Bee Farms, Sendirian Berhad. Geopropolis samples (10 kg) were ground and then extracted with 100% ethanol in Jiangsu Jiangdayuan Biology CO. LTD, to provide the EEGP (~3.8 kg).

Standards (all with purity ≥95%) of benzoic acid, syringic acid, chlorogenic acid, gallic acid, rosmarinic acid, caffeic acid, *p*-coumaric acid, cinnamic acid, ferulic acid, *trans*-isoferulic acid, 3,4-dimethoxycinnamic acid, rutin, quercetin, myricetin, luteolin, kaempferol, galangin, pinocembrin, hesperitin, chrysin, apigenin, morin, naringenin, pinobanksin, caffeic acid, phenethyl ester, and artepillin C were purchased from Heyuan (Shanghai, China) and Bingda Biology Co. (Beijing, China).

A stock solution (1 mg/mL) containing all standards was prepared and then diluted with methanol to obtain working standards at six different concentrations. The analytical stock standards were stored at −20 °C and working standards were stored at 4 °C.

### 3.2. UHPLC-Q-TOF/MS Analysis of EEGP

#### 3.2.1. Sample Preparation

The geopropolis sample collected from the whole honeycomb was simply crushed and washed with water to remove the carcass of the bees, sticks and other dirty things. Then the sample was extracted with ethanol and rest for one day, the extract was filtered through filter paper, centrifuged at 14,000× *g* for 10 min (TGL-20M, Changsha Xiangyi Centrifuge Instrument Co., Ltd., Changsha, China). The supernatants were combined, concentrated in rotary evaporator (Buchi R-215). About 5 mg of dried EEGP was dissolved in 1 mL 90% methanol (*v*/*v*) and passed through a 0.2-μm nylon membrane filter prior to UHPLC-Q-TOF/MS analysis.

#### 3.2.2. UHPLC System and Mass Spectrometry

UHPLC analysis was performed on an Agilent 1290 ultra-high performance liquid chromatography system (Agilent, Palo Alto, CA, USA) equipped with an Agilent ZORBAX SB-Aq C_18_ column (2.1 × 150 mm, 3.5 μm) at 30 °C. The mobile phase consisted of a linear gradient of 0.1% (*v*/*v*) aqueous formic acid (A) and methanol (B): 0–5.0 min, 1% B (*v*/*v*); 5.0–30.0 min, 1–30% B (*v*/*v*); 30.0–40 min, 30–95% B (*v*/*v*); 40.0–58.0 min, 95% B (*v*/*v*); 58.0–60.0 min, 95–1% B (*v*/*v*); 60.0–80.0 min, 1% B (*v*/*v*). The column was reconditioned for 5 min prior to the next injection. The flow rate was 0.3 mL/min, and the injected volume was 1 μL.

The MS analysis was performed on an Agilent 6545 Accurate-Mass Q-TOF/MS system with an electrospray ionization (ESI) source connected to the UHPLC. The ESI source parameters were: drying gas (N_2_); flow rate and temperature, 10.0 L/min and 350 °C; nebulizer, 40 psi; capillary voltages were 3500 V and 4000 V in negative and positive modes, respectively. The fragmentor voltage was 130 V in positive and negative modes. The collision energies were 40 V and 20 V in positive and negative MS/MS modes, respectively. The mass screening range was *m*/*z* 100–1500. All data were recorded and processed using the Agilent MassHunter Workstation software (Version B.04.00), Agilent MSC software (Version B.07.00) and the online METLIN database. The accuracy error threshold was set at ≤5 ppm.

In light of the advantages of UHPLC-Q-TOF/MS, the data acquisition mode of all target compounds is combined high resolution mass spectrometry with data dependent acquisition. To be specific, the mass spectra information for each constituent was obtained by selecting special precursor ions and collecting the corresponding fragment ions in a Quad Mass Filter and Collision Cell.

### 3.3. PHPLC Purification and NMR Analysis for Identification of Unknown Compounds in EEGP

#### 3.3.1. PHPLC

The 1260 PHPLC (Agilent, Waldbronn, Germany) consisted of a 1362 A preparative pump equipped with a G1365D multiple wavelength detector and a preparative column (Kromasil 100-5C18, 250 × 21.2 mm, 5 μm, Bohus, Sweden). The flow rate was set to 18 mL/min, the injection volume was 0.5 mL, and the column temperature was maintained at 30 °C. The mobile phase, elution conditions, and detection wavelength were the same as those used in the HPLC (Section 3.2.2). The sample was added to the column and the eluate containing the desired compound was reprocessed on the column several times until purified. Purified compounds were freeze-dried and analyzed using NMR.

#### 3.3.2. NMR

NMR spectra in CDCl_3_ were recorded on a Bruker AV III HD-400 instrument (Bruker, Karlsruhe, Germany) at 400 MHz for ^1^H and 100 MHz for ^13^C, using standard pulse programs and acquisition parameters. Chemical shifts are reported in δ (ppm) and referenced to the NMR solvent used.

## 4. Conclusions

In this study, a reliable and effective analytical method, based on UHPLC-Q-TOF/MS in combination with chemical structure prediction software, was developed for the rapid profiling and identification of compounds in EEGP produced by Malaysian stingless bees—*H. itama.* Using the online METLIN database and MSC software, 28 compounds were identified or tentatively identified in the ethanol extract. Some components were further confirmed based on authentic standards, in agreement with the tentative assignments made using the MSC software and the METLIN database. The results demonstrated that UHPLC-Q-TOF/MS, combined with a database and MS fragmentation analysis, was a simple and effective technology for the analysis of complex samples when some component standards were not available. Two abundant terpenoids in EEGP—24(*E*)-cycloart-24-ene-26-ol-3-one and 20-hydroxy-24-dammaren-3-one—were identified based on NMR and the literature data. These two components were identified for the first time in the geopropolis produced by *H. itama*, and are potential markers for this geopropolis. This comprehensive study provides essential data for further quality control, and for pharmacological and even toxicological studies of geopropolis produced by *H. itama*.

## Figures and Tables

**Table 1:** Identification of compounds in the ethanolic extract of geopropolis.

Peak	R_t_	*m*/*z* (−)	Error (ppm)	*m*/*z* (+)	Error (ppm)	Formula	MS/MS (*m*/*z*) (−)	MS/MS (*m*/*z*) (+)	Identification	Confirmation
1	2.2	169.0144	1.18	171.0283	−2.92	C_7_H_6_O_5_	125.0234, 107.0128	/	Gallic acid [17,30]	MSC and authentic standard
2	3.1	179.0342	−4.47	181.0489	−3.31	C_9_H_8_O_4_	135.0399,109.0301	/	Caffeic acid [18]	MSC and authentic standard
3	3.9	353.0876	−0.56	355.1019	−1.41	C_16_H_18_O_9_	191.0129, 179.0488, 173.0004, 161.0535, 154.9881	/	Caffeoylquinic acid [31]	MSC
4	4.6	331.1031	−1.21	333.1170	−3.00	C_14_H_20_O_9_	211.0224, 169.0133, 125.0232, 107.0127	/	Gallic acid-hexose [32]	MSC
5	5.5	167.0362	1.20	169.0491	−2.37	C_8_H_8_O_4_	153.0206, 108.0213	/	Vanillic acid [33]	MSC
6	7.9	167.0354	2.39	/		C_8_H_8_O_4_	153.0211, 108.0214	/	Isomer of vanillic acid [33]	MSC
7	9.3			195.0877	0	C_8_H_10_N_4_O_2_	/	150.0888, 138.0668, 135.0477, 110. 0713	Caffeine [34]	MSC
8	12.8	151.0402	0.66	153.0543	−1.96	C_8_H_8_O_3_	137.0233, 123.0463, 107.0120	/	Vanillin [33]	MSC
9	16.5	197.0452	−1.52	199.0611	5.02	C_9_H_10_O_5_	153.0481, 124.0161, 107.0477, 106.0062	/	Syringic acid [33]	MSC and authentic standard
10	21.8	153.0192	−0.65	155.0344	3.23	C_7_H_6_O_4_	123.0416, 109.0285	/	Protocatechuic acid [33]	MSC
11	25.2	125.0244	0	127.0391	0.79	C_6_H_6_O_3_	107.0130	/	Pyrogallol [33]	MSC
12	31.8	121.0293	−1.65	123.0440	−0.81	C_7_H_6_O_2_	105.0349	/	Benzoic acid	authentic standard
13	33.1	583.1118	4.29	/		C_28_H_24_O_14_	431.0997, 313.0578, 269.0467,169.0143, 125.0242	/	Vitexin-*O*-gallate [35]	MSC
14	34.0	583.1120	4.63			C_28_H_24_O_14_	431.0970, 313.0577, 269.0461, 169.0142, 125.0234	/	Isomer of vitexin- *O*-gallate [35]	MSC
15	34.9	205.0863	−3.41	207.1014	−0.97	C_12_H_14_O_3_	/	189.0526, 150.0297, 149.0232, 122.0335	Acetyleugenol [36]	MSC
16	36.8	271.0613	0.37	273.0754	−1.10	C_15_H_12_O_5_	229.0477, 211.0348, 187.0375, 151.0012	/	Pinobanksin	MSC and authentic standard
17	38.4	161.0243	−0.62	163.0396	3.68	C_9_H_6_O_3_	/	135.0447, 133.0286, 107.0512, 105.0347	Umbelliferone [37]	MSC
18	39.7	285.0417	4.21	287.0557	2.44	C_15_H_10_O_6_	255.0341, 239.0376, 227.0384, 211.0421, 199.0574, 124.0143, 107.0135	/	Kaempferol [33]	MSC and authentic standard
19	40.6	247.0972	−1.62	249.1123	0.80	C_14_H_16_O_4_	217.0884, 172.0888, 144.0939	/	Prenyl caffeate [38]	MSC
20	41.3	439.3583	0.23	441.3725	−0.45	C_30_H_48_O_2_	/	/	24(*E*)-cycloart-24-ene-26-ol-3-one [23]	NMR
21	42.4	241.0874	1.66	243.1017	0.41	C_15_H_14_O_3_	/	225.0560, 183.0806, 149.0597, 133.0665	Lapachol [39]	MSC
22	43.2	441.3736	−0.45	443.3882	−0.45	C_30_H_50_O_2_	/	/	20-hydroxy-24-dammaren-3-one [24]	NMR
23	44.9	407.1362	3.44	409.1488	−1.22	C_20_H_24_O_9_	245.0832, 230.0594, 202.0612, 187.0403, 173.0605, 137.0224	/	Torachrysone-*O*-hexose [30]	MSC
24	45.7	449.1462	2.00	451.1506	−0.66	C_22_H_26_O_10_	407.1322, 245.0482, 230.0065, 202.0516	/	Torachrysone-*O*-(acetyl)-hexose [30]	MSC
25	46.7	559.1467	1.79	561.1554	−1.60	C_27_H_28_O_13_	313.0575, 287.0938, 245.0827, 230.0596, 215.0367, 169.0143, 125.0232	/	Torachrysone-*O*-(galloyl)-hexose [30]	MSC
26	47.8	409.1656	−0.24	411.1801	−0.24	C_24_H_26_O_6_	/	393.1688, 355.1167, 341.1009, 299.0541, 195.1013	Mangostin [40]	MSC
27	48.7	437.3424	−0.23	439.3570	−0.23	C_30_H_46_O_2_	/	421.3449, 249.1849, 235.1698, 167.1074	Ganoderol A [41]	MSC
28	50.0	427.3582	0	429.3712	−3.93	C_29_H_48_O_2_	/	411.3611, 193.1608	Saringosterol [42]	MSC
29	52.3	411.3629	−0.73	413.3757	−5.08	C_29_H_48_O	/	395.3651, 135.1171	Stigmasterol [43]	MSC
30	56.0	423.3630	−0.47	425.3777	−0.24	C_30_H_48_O	/	407.3648, 271.2086, 135.1169, 109.1019	Taraxerone [44]	MSC

Note: “R_t_” indicates retention time; “−” indicates negative mode; “+” indicates positive mode; “MSC” means Molecular Structure Correlator.

## Supplementary Materials

Supplementary File 1
