# Supplementary figures and images for: Rapid Determination of Major Compounds in the Ethanol Extract of Geopropolis from Malaysian Stingless Bees, Heterotrigona itama, by UHPLC-Q-TOF/MS and NMR
Source: Molecules. 2017 Nov 10;22(11):1935. doi: 10.3390/molecules22111935 (PMC6150372; doi:10.3390/molecules22111935)

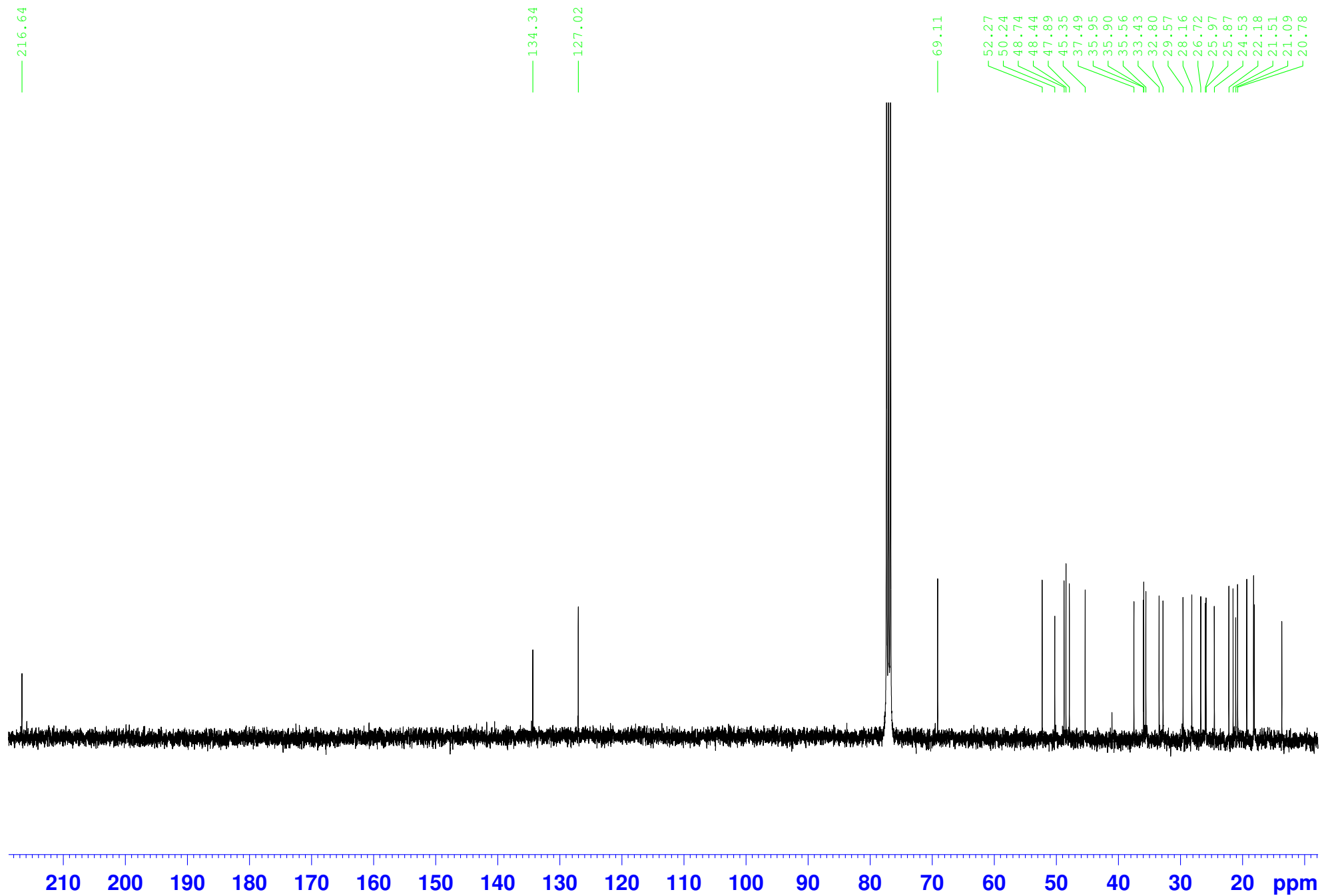

Supplement: Supplementary File 1 [file molecules-22-01935-s001.zip › The Data of Nuclear Magnetic Resonance/13 C NMR spectral data of 24(E)-cycloart-24-ene-26-ol-3-one.pdf]

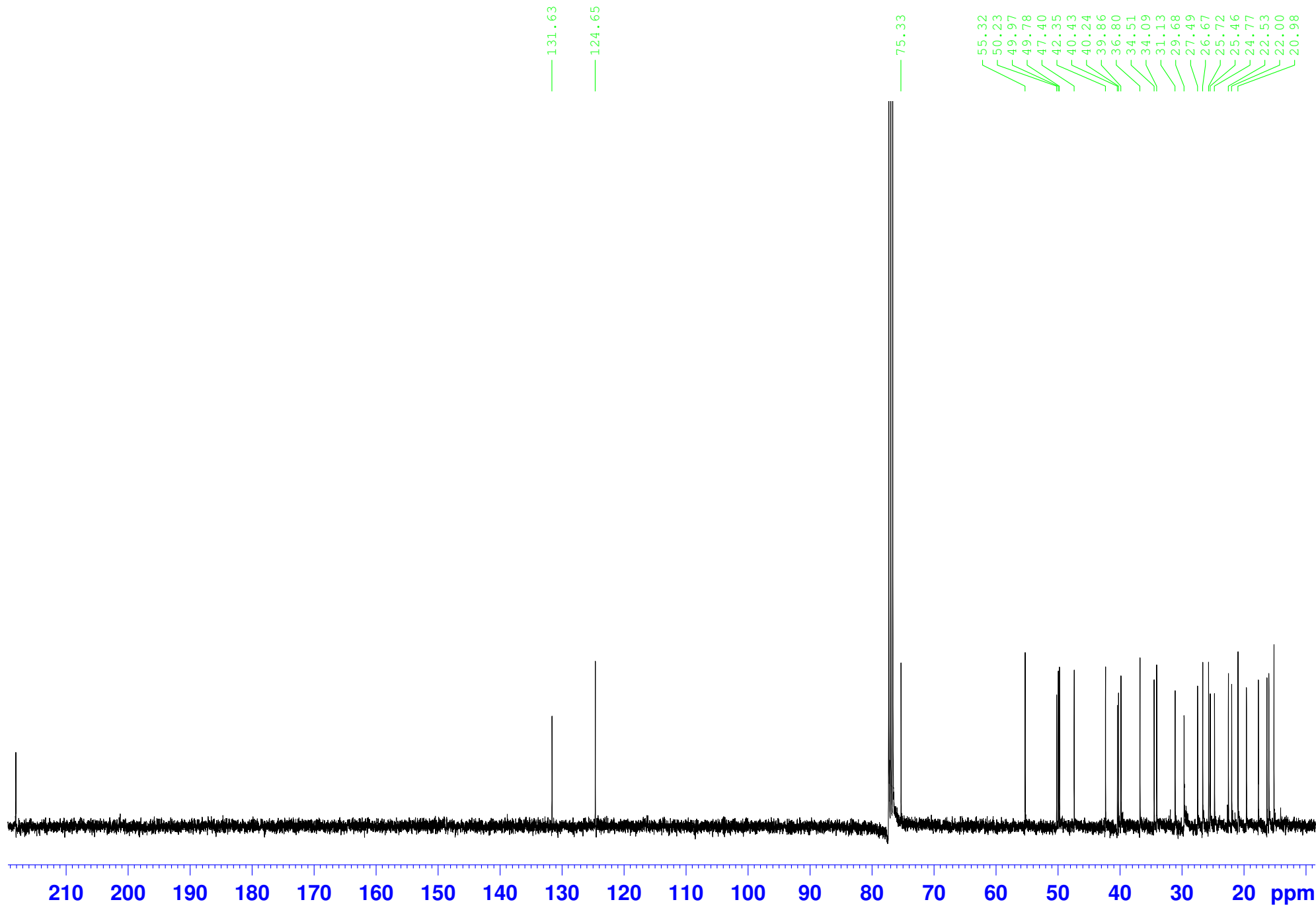

Supplement: Supplementary File 1 [file molecules-22-01935-s001.zip › The Data of Nuclear Magnetic Resonance/13C NMR spectral data of 20-hydroxy-24-dammaren-3-one.pdf]

H1 2

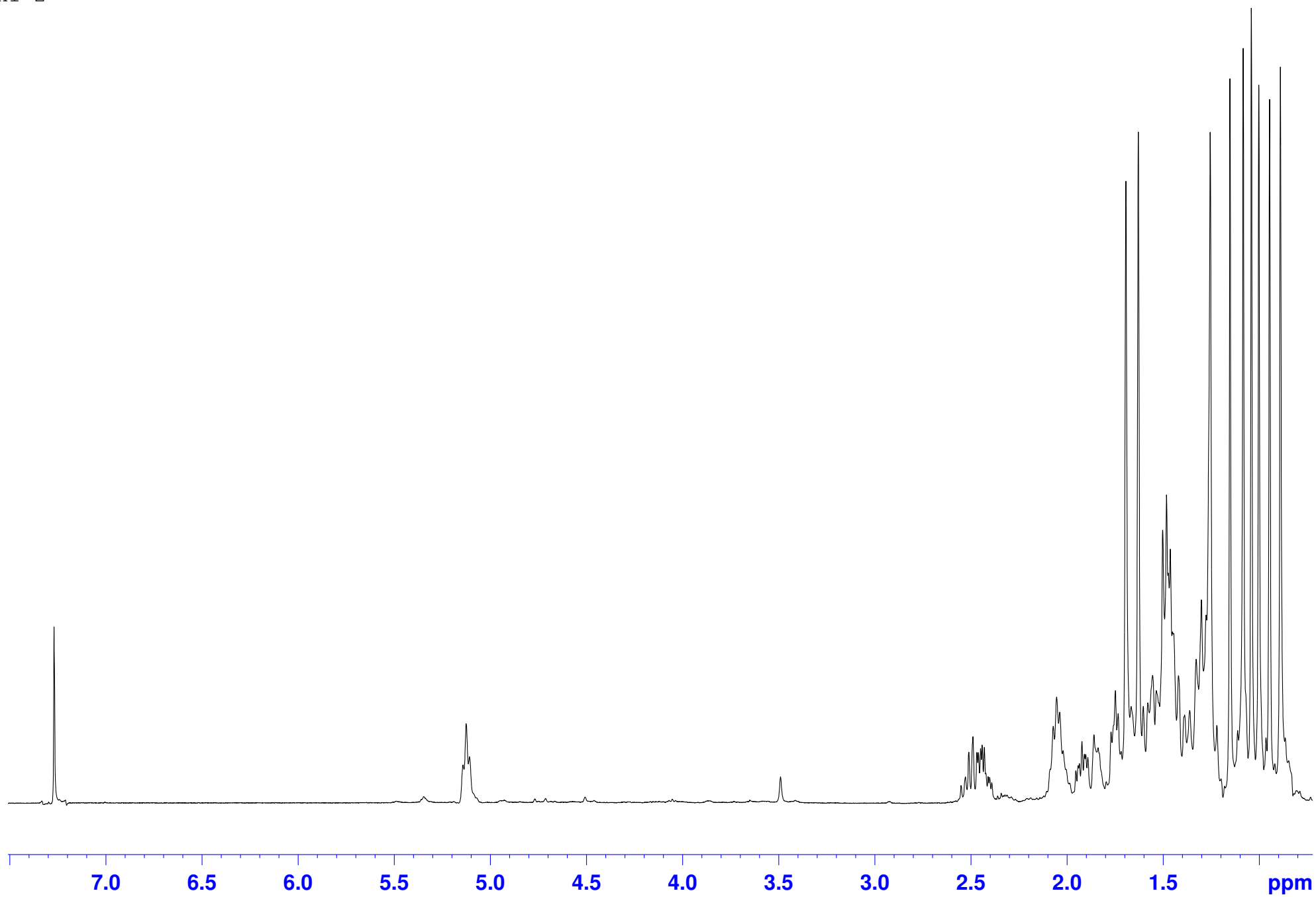

Supplement: Supplementary File 1 [file molecules-22-01935-s001.zip › The Data of Nuclear Magnetic Resonance/1H NMR spectral data of 20-hydroxy-24-dammaren-3-one.pdf]

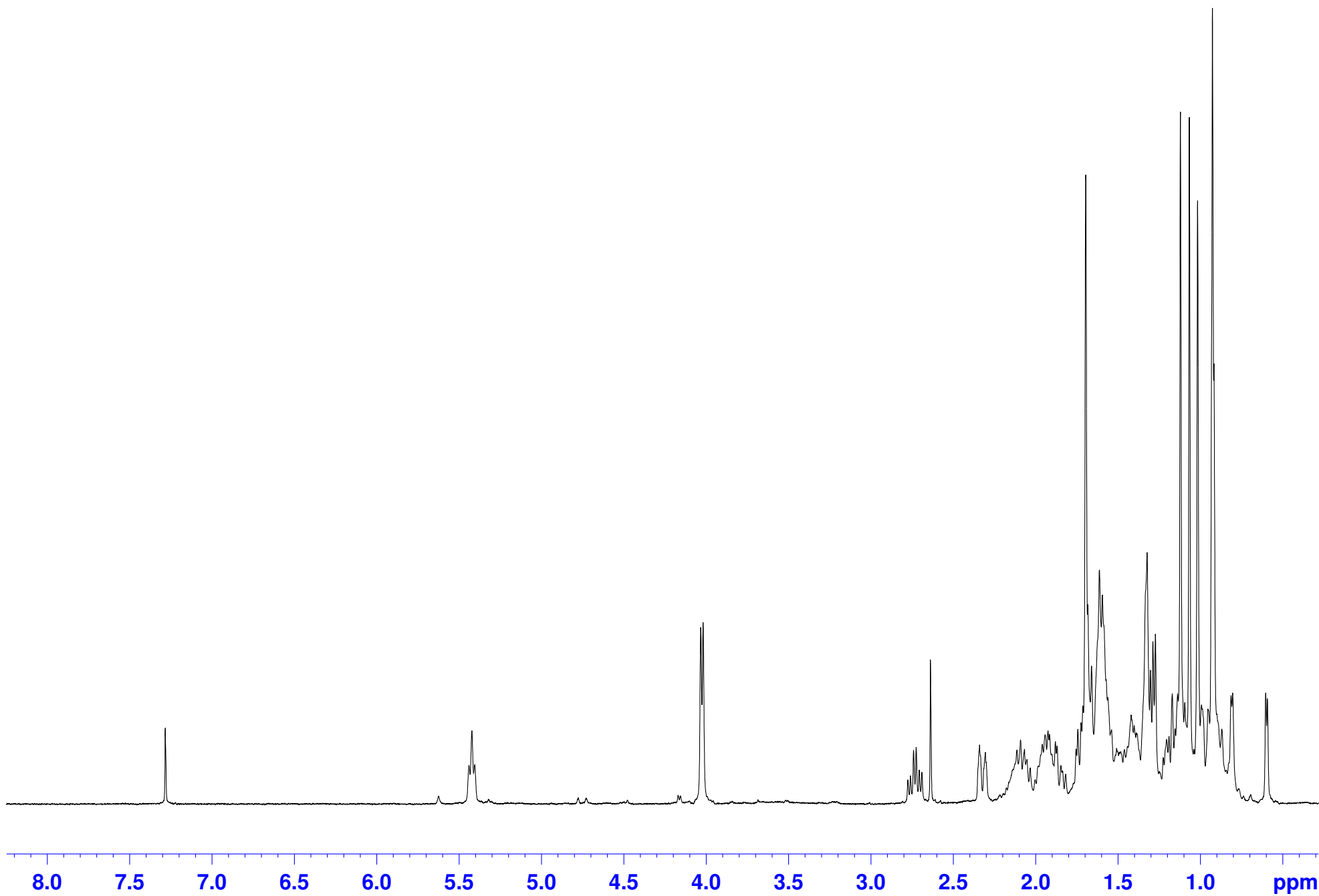

Supplement: Supplementary File 1 [file molecules-22-01935-s001.zip › The Data of Nuclear Magnetic Resonance/1H NMR spectral data of 24(E)-cycloart-24-ene-26-ol-3-one.pdf]
